# A G-quadruplex DNA structure resolvase, RHAU, is essential for spermatogonia differentiation

**DOI:** 10.1038/cddis.2014.571

**Published:** 2015-01-22

**Authors:** X Gao, W Ma, J Nie, C Zhang, J Zhang, G Yao, J Han, J Xu, B Hu, Y Du, Q Shi, Z Yang, X Huang, Y Zhang

**Affiliations:** 1 MOE Key Laboratory of Model Animal for Disease Study, Nanjing Biomedical Research Institute, National Resource Center for Mutant Mice, Model Animal Research Center of Nanjing University, Nanjing 210061, China; 2 Shanghai Key Laboratory for Molecular Andrology, State Key Laboratory of Molecular Biology, Institute of Biochemistry and Cell Biology, Shanghai Institutes for Biological Sciences, Shanghai 200031, China; 3 Hefei National Laboratory for Physical Sciences at Microscale, School of Life Sciences, University of Science and Technology of China, Hefei, 230026 Anhui, China; 4 Shanghai Institute of Planned Parenthood Research, Shanghai 200032, China

## Abstract

G-quadruplex (G4) DNA and G4 DNA resolvase are involved in a variety of biological processes. To understand the biological function of G4 DNA structures and their resolvases in spermatogenesis, we investigated the distribution of G4 structures in mouse testis and identified their alterations during spermatogenesis. Meanwhile, we studied the function of RNA helicase associated with AU-rich element (RHAU), a G4 DNA resolvase, in spermatogenesis with a germ-cell-specific knockout mouse model. The results showed that the ablation of RHAU in germ cells caused the increase of G4 structures and thus resulted in the decrease of spermatogonial differentiation. c-kit, a spermatogonia differentiation-related gene, contains two G4 DNA motifs on its promoter. We found its expression was significantly downregulated in RHAU conditional knockout testis. A further analysis demonstrated that RHAU directly bound to the G4 structures to activate c-kit expression. We concluded that RHAU regulates spermatogonia differentiation by promoting c-kit expression via directly binding to the G4 DNA motifs c-kit promoter.

G-quadruplex (G4) structures are stacked nucleic acid structures that can form within specific repetitive G-rich DNA or RNA sequences.^1, 2^ In these tetramers, four guanine molecules form a square planar arrangement in which each guanine is hydrogen bonded to the two adjacent guanines.^2^ G4 structure is stabilized by monovalent cations that occupy the central cavities between the stacks, neutralizing the electrostatic repulsion of inwardly pointing guanine oxygens.^3, 4^ In 1962, *Gellert* and colleagues discovered the tetrameric structures *in vitro* using X-ray diffraction.^5^ Recently, an intriguing finding has emerged that G4 DNA structures in mammalian cells can be directly visualized through the use of a highly specific antibody developed by two research groups,^6, 7^ corroborating that G4 structures truly exist *in vivo*. Bioinformatic analyses revealed the prevalence of G4 motifs in key regulatory regions of the human genome, such as promoters, gene bodies, untranslated regions, and the ends of chromosomes (telomeres).^8, 9, 10, 11^ The emerging evidences suggest that G4 DNA structures have crucial roles in a variety of biological processes, including transcription, recombination, replication, and maintaining chromosome stability.^2, 12, 13, 14, 15^

Given the fact that G4 DNA structures participate in essential biological processes, the formation of these structures is likely to be actively modulated during the embryogenesis and differentiation in a similar way as the regulation of DNA and histone methylation. Generally, G4 DNA structures are highly stable. However, the precisely spatial and temporal assembly and disassembly of these structures require the G4 DNA chaperones and resolvases. To rapidly remove G4 DNA structures, resolvases initiate the process through recognizing and resolving them. To date, several resolvases have been observed to catalyze the resolution of G4 DNA structures back to the single-stranded DNA, such as viral SV40 large T-antigen helicase,^16^ human Werner's syndrome (*WRN*), and Bloom's syndrome (*BLM*) helicase,^17^ *DHX36,*^18, 19^ *DHX9,*^20^ *DDX11,*^21^ *RTEL1,*^22^ and *PIF1.*^23^ The deficiency of these resolvases often leads to embryonic lethality, genomic instability, unintegrated telomere, and senescence.^24, 25, 26, 27, 28^ Thus, the functional regulation G4 DNA resolvases appears to be indispensable in the normal development and physiological functions of various types of cells.

The process of spermatogenesis can be subdivided into three major steps: (1) mitotic cell division and differentiation, (2) meiosis initiation and progresses, and (3) spermatid deformation. We hypothesized that G4 DNA structures and their resolvases have an important role in spermatogenesis. And, it has been shown that in *Saccharomyces cerevisiae*, G4-DNA-related chaperon and resolvase are involved in the meiotic recombination.^29, 30^ However, it is not clear whether G4-DNA itself is directly related to the meiotic process. Therefore, the roles of G4 DNA and its binding proteins in gametogenesis of mammalian system still need to be investigated.

RNA helicase associated with AU-rich element (RHAU; alias DHX36, G4R1), a highly conserved gene, is highly expressed in human testis.^31^ Previous studies revealed that RHAU could unwind the G4 structures of DNA and RNA.^19, 32^ RHAU ablation caused embryonic lethality at ~7.0 days postcoitus (d.p.c.); and specific deletion of RHAU in hematopoietic system affected the development of several lineages. Both of them indicated that RHAU is essential for the early embryonic development and hematogenesis.^33^ In this paper, we generate germ-cell-specific RHAU knockout mice to investigate the function of RHAU in spermatogenesis. We find that the male germ-cell-specific ablation of RHAU could bind G4-DNA structures directly and unwind them with ATP, leading to spermatogenesis failure. These results demonstrate the critical and direct roles of G4 DNA and RHAU resolvase in spermatogenesis.

## Results

### RHAU expressed at multiple germ cell stages

**Figure 1:**
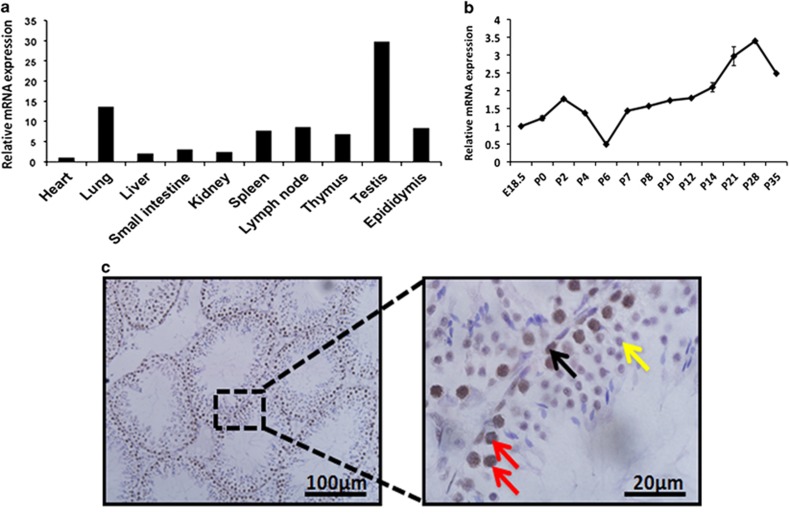
RHAU expression pattern in mouse testes. (**a**) Quantitative PCR analysis of the RHAU expression in multiple tissues. (**b**) Quantitative PCR analysis of the RHAU expression in whole testes at different stages of development. (**c**) Immunohistochemistry assay of the RHAU expression (brown) in testes of P35 mice. The section was counterstained by hematoxylin (blue). Black arrow represents spermatogonial stem cell (SSC), red ones represent primary spermatocytes and yellow arrow represents round spermatid. The magnification, left: × 20, right: × 100

It has been previously reported that RHAU was highly expressed in human testis.^31^ To study if RHAU regulates mouse spermatogenesis, we first tested the expression of RHAU in mouse tissues using reverse transcription and quantitative PCR (RT-qPCR). We found that RHAU was expressed at a higher level in mouse testis when compared with other tissues: heart, lung, liver, kidney, small intestine, epididymis, and immune organs, such as spleen, lymph nodes, and thymus (Figure 1a). Next, we investigated the expression of RHAU at different developmental stages of mouse testis. By RT-qPCR analysis, we found that RHAU mRNA displayed a dynamic change during the testicular development from embryonic day 18.5 to postnatal day 35 (P35; Figure 1b). RHAU mRNA was least expressed at P6, and a maximum level of expression was seen at P28 (Figure 1b). Using immunohistochemical analysis of RHAU protein distribution in different cell types in adult mouse testis, we found that RHAU was highly expressed in spermatogonia stem cells (SSCs) and primary spermatocytes, but lowly expressed in spermatids (Figure 1c). These data suggested RHAU likely played various roles at different stages of spermatogenesis.

### Specific RHAU deletion in germ cells leads to azoospermia

**Figure 2:**
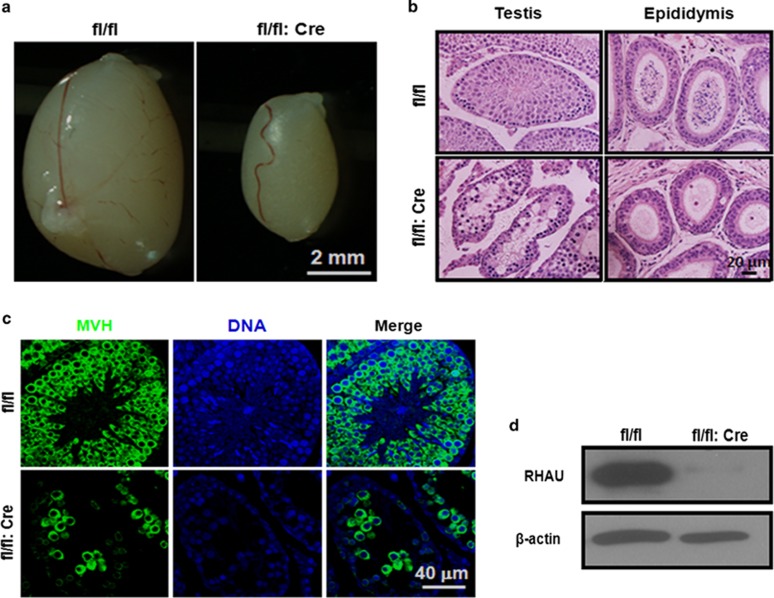
RHAU deletion resulted in the failure of meiosis. (**a**) Representative images of testes from P35 control and RHAU deletion mice. (**b**) Representative H&E staining of testes from P35 control and RHAU deletion mice. (**c**) Representative images of immunostaining of MVH (green) of testes from P35 control and RHAU deletion mice. Cell nuclei were stained with DAPI (blue). (**d**) Western blotting assays of the RHAU expression in whole testes from P35 control and RHAU deletion mice

To dissect the function of RHAU in spermatogenesis, we generated germ-cell-specific RHAU knockout mouse model by crossing RHAU floxed mice with Vasa-Cre transgenic mice (the Vasa promoter-driven Cre expression was efficiently and specifically in germ cells beginning at 15.5 d.p.c.^34^). By using the western blot analysis, we demonstrated that the protein level of RHAU was dramatically decreased at P35 (Figure 2d). The germ cell-specific deletion of RHAU developed testicular hypoplasia (Figure 2a). RHAUfl/fl:Vasa-Cre mice displayed a smaller testis at P35 (Figure 2a). To characterize the spermatogenesis failure, standard hematoxylin and eosin (H&E) staining was used to detect the status of mature sperm in testis and epididymal cauda. The staining showed that mature sperm was absent in the epididymis cauda of RHAU deletion mice (Figure 2b). Immunofluorescence analysis against a germ cell marker, MVH (Ddx4/Vasa), further confirmed the absence of germ cells (Figure 2c). Thus, the deletion of RHAU specifically in germ cells resulted in azoospermia.

### Meiotic failure in RHAU-deleted mice

**Figure 3:**
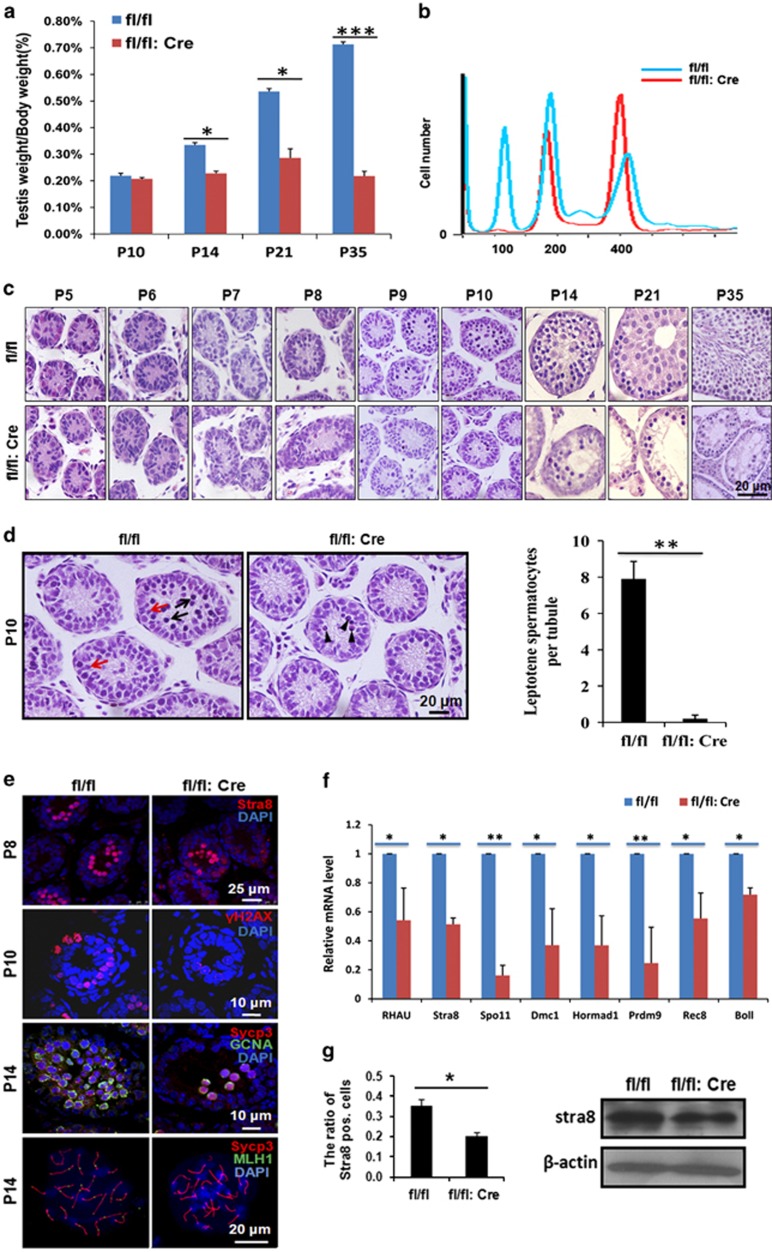
Germ cell-specific RHAU knockout decreased meiotic cells dramatically. (**a**) Testes/body weight of P10, P14, P21, and P35 control and RHAU deletion mice (mean±S.D., *n*=3; *P<0.05, ***P<0.001). (**b**) Flow cytometry analysis of DNA contents of germ cells from P21 control and RHAU deletion mice. (**c**) Representative H&E staining of testes from P5, P6, P7, P8, P9, P10, P14, P21, and P35 control and RHAU deletion mice. (**d**) Representative zoomed-in images of H&E staining of testes from P10 control and RHAU deletion mice (left). Red arrows indicate preleptotene spermatocytes, and the black arrows indicate the leptotene spermatocytes in the control mice. The black arrow heads show the apoptotic cells in the mutant mice. The analysis of cell counting showed the decreased leptotene spermatocytes in RHAU knockout testis (mean±S.D., *n*=3; **P<0.01; right). (**e**) Co-immunostaining of Stra8 (red; upper), *γ*H2 AX (red; middle), and Sycp3 (red; lower) in P8, P10, and P14 control and RHAU ablation testes, respectively. GCNA-positive cells (green) represent germ cells. Spermatocyte spreading analysis of Sycp3 (red) and MLH1 (green) in P14 wild-type and RHAU ablation testis. (**f**) Quantitative PCR analysis of mRNA levels of RHAU, Stra8, Spo11, Dmc1, Hormad1, Prdm9, Rec8, and Bo11 in whole testes from P8 control and RHAU deletion mice (mean±S.D., *n*=3; *P<0.05, **P<0.01). (**g**) The percentage of Stra8-positive cells in testes from P8 control and RHAU deletion mice (left; mean±S.D., *n*=5; *P<0.05). Western blot analysis of Stra8 expression in whole testes from P8 control and RHAU mice (right)

To further characterize the abnormal spermatogenesis in the RHAUfl/fl:Vasa-Cre mice, we measured testes weight during the postnatal development and revealed that the testes significantly lost weight from P14 to P35 (Figure 3a), indicating the spermatogenesis process might be disrupted as early as meiosis I stage. Consistently, flow cytometry analysis of P21-testes-derived cells revealed that tetraploid, not haploid, cells were present in RHAUfl/fl:Vasa-Cre mice (Figure 3b). Subsequently, the testes of wild-type and RHAU-ablated mice at different developmental time points were sectioned and subjected to H&E staining. Apparently, there were some types of spermatocyte deficiency in testes of P14 RHAU knockout mice (Figure 3c). Moreover, the percentage of leptotene spermatocytes in RHAU knockout testes was significantly decreased (Figures 3c and d), indicating that the meiosis failure might be caused by the early disruption of meiosis initiation, spermatogonia differentiation, or even SSC self-renewal. Consistent with the above observation, the number of cells, which were positive for *γ*H2AX (a DNA double-stranded break marker^35^), Sycp3 (a main component of synaptonemal complex^36^) and MLH1 (a crossover associated gene^37^), were dramatically decreased in RHAU knockout testes (Figure 3e, middle, lower, and the fourth line panel). The expression of a series of meiosis-related genes, such as Hormad1,^38^ Dmc1,^39^ and Spo11,^40^ were all significantly downregulated in testes of P10 RHAU knockout mice (Figure 3f). All these evidences demonstrated that RHAU deletion disrupted meiosis initiation.

Next, we tested the expression of Stra8, a gene essential for meiosis initiation,^41^ in RHAUfl/fl:Vasa-Cre mice. Immunofluorescence analysis results showed a decrease in Stra8 staining cells in P8 testes (Figure 3g (left) and the upper panel of Figure 3e). Consistently, qPCR and western blotting assays revealed Stra8 mRNA and protein levels were also decreased in Figures 3f and g (right), respectively. Thus, the ablation of PHAU in germ cells may downregulate Stra8 expression and result in meiosis initiation blockage.

### Spermatogonia self-renewal unaffected by the decreased differentiation of spermatogonia in RHAU-deleted mice

**Figure 4:**
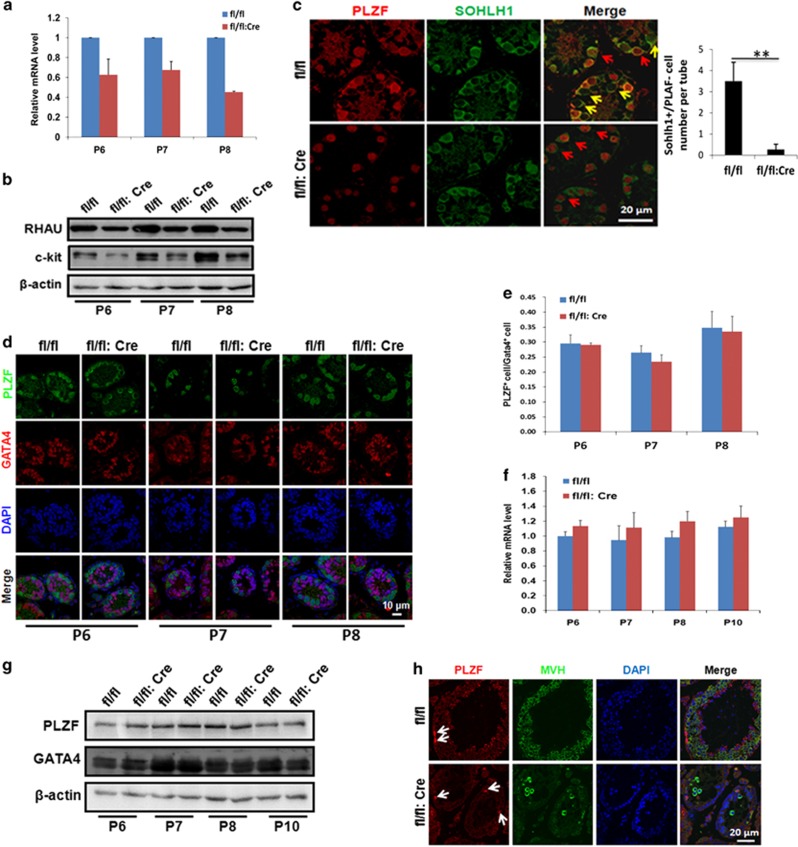
Germ cell-specific RHAU knockout led to decreased spermatogonia differentiation without affecting SSC development. (**a**) Quantitative PCR analysis of *c-kit* expression in whole testes of P6, P7, and P8 control and RHAU deletion mice (mean±S.D., *n*=3). (**b**) Western blot analysis of *c-kit* expression in whole testes of P6, P7, and P8 control and RHAU deletion mice. (**c**) Co-immunostaining of PLZF (red) and SOHLH1 (green) of testes from P8 control and RHAU deletion mice. Yellow arrows represented SOHLH1-positive cells, whereas red arrows indicated PLZF and SOHLH1 co-staining-positive cells. The right panel shows the result of the counting of SOHLH1-positive and PLZF-negative spermatogonia (mean±S.D., *n*=3; ** *P*<0.01). (**d**) Representative images of co-immunostaining of PLZF (green) and GATA4 (red) of testes from P6, P7, and P8 control and RHAU deletion mice. (**e**) The ratio of PLZF-positive cells to GATA4-positive cells in testes from P6, P7, and P8 control and RHAU deletion mice (mean±S.D., *n*=4). (**f**) Quantitative PCR analysis of PLZF expression whole testes from P6, P7, P8, and P10 control and RHAU deletion mice (mean±S.D., *n*=3). (**g**) Western blot analysis of PLZF and GATA4 expression in whole testes from P6, P7, P8, and P10 control and RHAU deletion mice. (**h**) Representative images of co-immunostaining of PLZF (red) and MVH (green) of testes from P62 control and RHAU deletion mice. The white arrows represent PLZF-positive cells. (**d** and **h**) DAPI staining of the same sections

The full differentiation of spermatogonia is essential for meiosis initiation.^41, 42, 43^ Therefore, we next studied differentiating spermatogonia in testes of RHAU knockout mice. As c-kit is a differentiating spermatogonia marker,^42^ we performed qPCR and western blot assays to detect its transcription and translation activities. The results showed that the mRNA and protein levels of c-kit were consistently downregulated in testes of RHAU-deleted mice (Figures 4a and b). Furthermore, we assessed the expression of other differentiation markers important in spermatogenesis. Immunofluorescence assays of SOHLH1 (e.g., another differentiating spermatogonia marker^44^) and PLZF (e.g., a marker of SSC^45, 46^) demonstrated that SOHLH1-positive cells expressed on their own in wild-type mice, as well as co-localized with PLZF-positive cells. Nevertheless, in RHAU-deleted mice, all the SOHLH1-positive cells overlapped with PLZF-positive ones. Therefore, more SOHLH1-positive cells were observed in testes of RHAU wild-type mice (Figure 4c), further suggesting a decrease in differentiating spermatogonia in the testes of RHAU-deleted mice. Taken together, these data strongly indicated that RHAU deletion led to insufficient differentiation of spermatogonia, resulting in the failure of meiotic initiation and progression.

We further investigated whether RHAU deletion would impair undifferentiated spermatogonia. We speculated that germ cell-specific deletion of RHAU could not apparently alter the number of Sertoli cells, as the number of Sertoli cells in wild-type mice did not differ from that in RHAU deletion mice (data not shown). Therefore, we analyzed PLZF-positive cells using co-immunofluorescence staining with GATA4, a marker of Sertoli cells.^47^ As a result, the PLZF-positive cells did not show any apparent changes (Figures 4d and e) in testes of P6, P7, and P8 RHAU knockout mice. Subsequent qPCR and western blot assays further confirmed there were no detectable PLZF changes at both mRNA and protein levels in testes of P6, P7, P8, and P10 RHAU knockout mice (Figures 4f and g). Then, we raised a question that whether the decrease of undifferentiated spermatogonia was accompanied with the postnatal development. To address this question, we analyzed the PLZF-positive cells in testes of P62 mice by immunofluorescence assay. The results showed that when compared with wild type, the PLZF-positive cells also did not exhibit an apparent decrease in RHAU-deleted testes (Figure 4h). Thus, it seems that germ cell-specific RHAU knockout has no effect on the undifferentiated spermatogonia. To further confirm this result, we detected another two marker genes of undifferentiated spermatogonia, Lin28^48^ and GFR-*α.*^49^ Consistently, their expression did not alter obviously (Supplementary Figures 1A and B). Taken together, germ cell-specific deletion of RHAU does not affect undifferentiated spermatogonia self-renewal, but it does influence differentiated spermatogonia development.

### RHAU deletion reducing proliferation and enhancing apoptosis of spermatogonia

**Figure 5:**
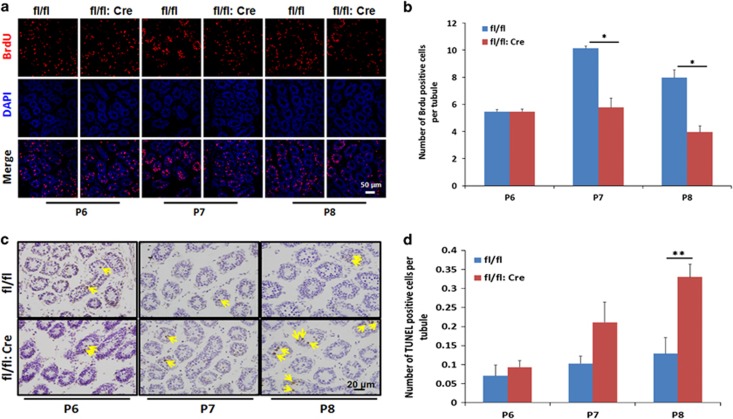
RHAU deficiency in germ cells results in decreased cell proliferation and increased apoptosis of spermatogonia. (**a**) Representative images of BrdU *in vivo* labeling showed proliferating cells (red) and DAPI counterstaining (blue) in testes sections from P6, P7, and P8 control and RHAU deletion mice. (**b**) The number of BrdU-positive cells per tubule in testes from P6, P7, and P8 control and RHAU deletion mice (mean±S.D., *n*=3; *P<0.05). (**c**) TUNEL assay of apoptosis cells (brown) and hematoxylin counterstaining (blue) in testes from P6, P7, and P8 control and RHAU deletion mice. The yellow arrows represent TUNEL-positive cells. (**d**) The number of apoptosis cells per tubule in testes from P6, P7, and P8 control and RHAU deletion mice (mean±S.D., *n*=3; **P<0.01)

The fact that RHAU deletion leads to no apparent change in undifferentiated spermatogonia, but significant decreases in differentiating cells, suggests possible abnormal cell proliferation and/or cell death during spermatogonia differentiation. To verify this possibility, we first monitored *in vivo* proliferation of spermatogonia in P6, P7, and P8 testes by intra-peritoneal injection of the DNA analog 5-bromo-2-deoxyuridine (BrdU; see 'BrdU incorporation assay'). Two hours after injection, the testes were sectioned and analyzed. As shown in Figures 5a and b, there was no apparent change of BrdU-positive cell population between wild-type and RHAU deletion testes in P6. However, the BrdU-positive cell population declined significantly in P7 and P8 RHAU deletion testes (*P*<0.05), indicating downregulated cell proliferation. Meanwhile, we tested whether RHAU ablation led to the elevation of cell death of spermatogonia by using terminal deoxynucleotidyl transferase dUTP nick end labeling (TUNEL) assay. The results showed a dramatical increase in apoptotic spermatogonia in mutant testes at P8, but not at P6 and P7 (Figures 5c and d). These data suggested that the differentiation blockage of the RHAU-ablated spermatogonia might be caused by the reduced proliferation and enhanced apoptosis.

### RHAU deficiency leading to the differential expression of a set of cell differentiation-related genes

**Figure 6:**
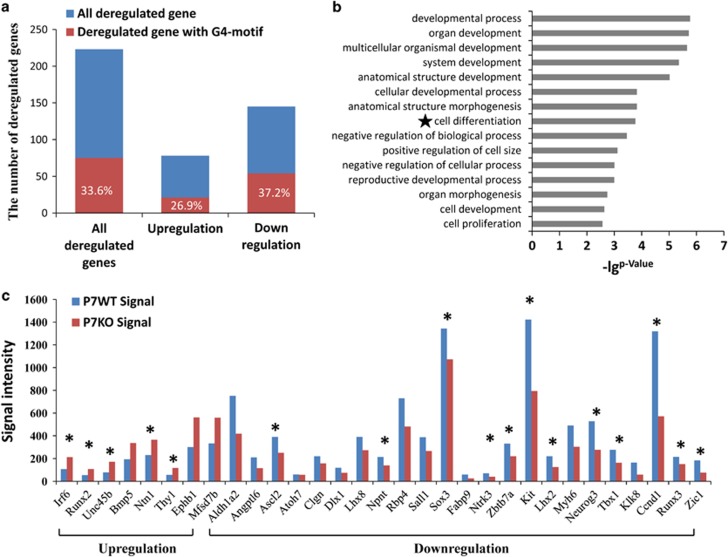
The deregulated genes in RHAU ablation testis. (**a**) The number of total deregulated genes, and G4 DNA-related deregulated genes in testes of P7 control and RHAU deletion mice. The red bars represent the number of G4 DNA-related deregulated genes, and meanwhile, the blue ones represent all deregulated genes. (**b**) Gene ontology assay of P7 deregulated genes by using DAVID tools. The differentially expressed genes associated with cell differentiation showed significant changes. (**c**) The differentially exrepessed genes with cell differentiation in P7 testes. The asterisks label the G4 DNA-related genes. *P<0.001

To explore the reason behind RHAU knockout-mediated reduction of differentiated spermatogonia, we examined the effect of RHAU ablation on gene expression in testes of P7. Transcriptomes of the testes from wild-type and germ cell-specific RHAU deletion mice were obtained by microarray analyses. A list of differentially expressed genes was generated with a cutoff of 1.5-fold change. There were 223 differentially expressed genes (75 genes with G4 motif, 33.6%), of which 78 were upregulated (21 genes with G4-DNA motif, 26.9%) and 145 were downregulated (54 genes with G4-DNA motif, 37.2% Figure 6a). Gene ontology analysis revealed these differentially expressed genes were associated with development, especially cell differentiation (Figure 6b). Thirty-one differential expressed genes enriched in the pathway of the cell differentiation, with eight of them being upregulated, whereas twenty-three of them downregulated. Among these cell differentiation-associated genes, there were 17 genes with G4-DNA motif (54.8%), indicating that RHAU may regulate cell differentiation by acting on the G4-DNA structures on the promoter of cell differentiation-related genes (Figure 6c).

### RHAU directly binding to G4 DNA motif on the promoter region of *c-kit* to enhance *c-kit* expression

**Figure 7:**
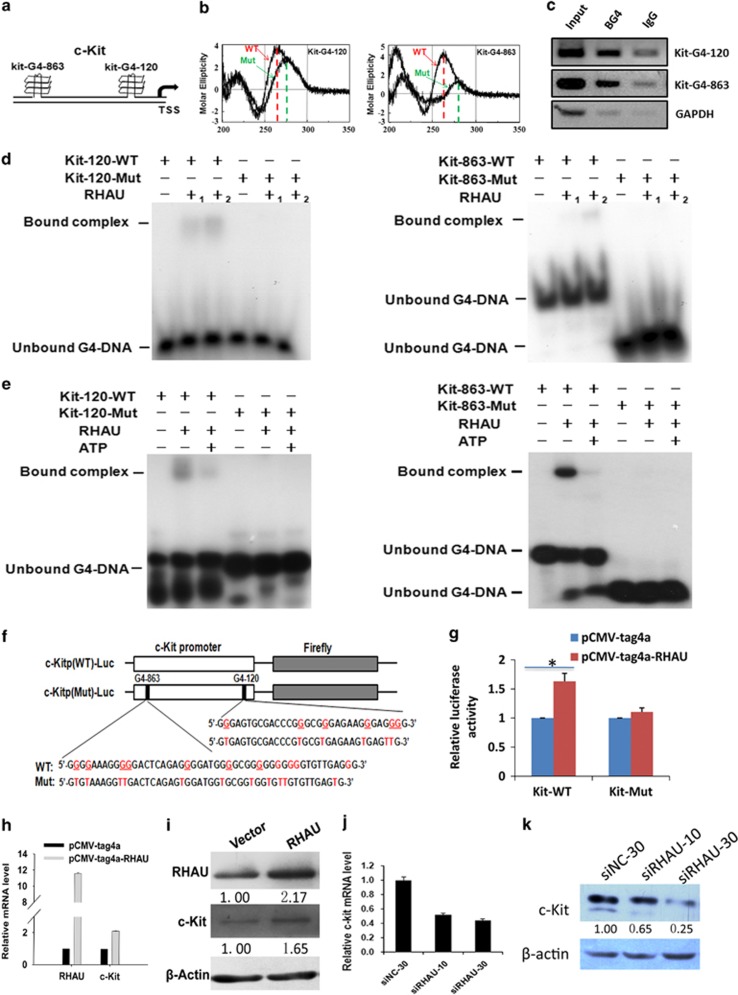
RHAU binds to the G4 DNA motif, which was unwound by ATP on the promoter of *c-kit* and directly regulates *c-kit* expression. (**a**)There are two putative G4 DNA motifs locating on the promoter of mouse *c-kit* gene, Kit-G4-120 and Kit-G4-863. (**b**) *In vitro* CD analyses of the oligodeoxyribonucleotides. The red dashed lines (red arrows) indicate the signature peaks of parallel G4 structure at 262 nm, whereas the green dashed lines (green arrows) indicate the peaks of molar ellipticity after the G4 structure-forming sequences were mutated. (**c**) Input sample and ChIP samples of BG4 and DYKDDDDK Tag antibody or normal IgG were analyzed by PCR to confirm ChIP-seq results at the target loci. BG4 could be enriched at the sites of Kit-120 and Kit-863. GAPDH was used as the negative control. (**d**) Gel mobility shift assay of individual wild-type G4 DNA structures bound by RHAU on promoters of *c-kit* and mutated G4 DNA structures at 120 bp (left) and 863 bp (right). The position of G4 DNA complexes and the unbound G4 DNA are denoted on the left. An amount of 1 pM of 5′-^32^P-labeled self-annealed Kit-120-WT (lanes 1–3) and Kit-120-Mut (lanes 4–6) were incubated with increasing amounts of purified RHAU ('-' represented 0, '+_1_' represented 1 *μ*g, '+_2_' represented 2 *μ*g), as indicated on the top. In the right panel, 1 pM of 5′-^32^P-labeled self-annealed Kit-863-WT (lane 1-3) and Kit-863-Mut (lanes 4–6) denoted the same as the left panel. (**e**) EMSA-detected ATP-dependency of RHAU/*c-kit* association, which binds and resolves G4-DNA structures at promoters of *c-kit* and mutated ones at 120 bp (left) and 863 bp (right). The position of G4 DNA complexes and the unbound G4 DNA are denoted on the left. An amount of 1 pM of 5′-^32^P-labeled self-annealed Kit-120-WT (lanes 1–3) and Kit-120-Mut (lanes 4–6) were incubated with purified RHAU ('-' represented 0, '+' represented 2 *μ*g), and ATP ('-' represented 0, '+' represented 5 mM), as indicated on the top. Kit-863 of the right panel is similar to Kit-120 of the left one. (**f**) Representative schematic diagrams of the constructs generated for reporter assays. (**g**) Luciferase assay of the effects of RHAU on luciferase expression mediated by wild-type (left) and double-mutated (right) c-kit promoter. RHAU-expressing plasmid or the empty vector were co-transfected HeLa cells with either the reporter construct containing wild-type c-kit promoter sequence, or mutated c-kit promoter sequence in 96-well plate and 24 h later, the luciferase activity was analyzed using double luciferase assay kit (*P<0.05). (**h**) Quantitative PCR analyses of *c-kit* mRNA level in HEK 293T cells 48 h after transfection with pCMV-tag4 vector and pCMV-tag4-RHAU vector. (**i**) Western blot analysis of RHAU and *c-kit* expression in HEK 293T cells 48 h after transfection with pCMV-tag4 vector and pCMV-tag4-RHAU vector. (**j**) Quantitative PCR analyses of *c-kit* mRNA level in HEK 293T cells 48 h after transfection with siRNA against RHAU at the concentrations of 10 nM and 30 nM, respectively. (**k**) Western blotting analyses of c-kit protein level in HEK 293T cells 48 h after transfection with siRNA against RHAU at the concentrations of 10 nM and 30 nM, respectively

*c-kit* is one of the differentiation-related genes harboring putative G4 motif, regulating the proliferation and differentiation of spermatogonial stem cells. It mutates leading to spermatogonia differentiation block, meiosis initiation arrest, decreased cell proliferation, and elevated cell apoptosis,^42, 50, 51, 52^ which are similar to the defects of RHAU deletion. qPCR and western blot analyses confirmed that *c-kit* was dramatically downregulated in testes of RHAU deletion mice (Figures 4a and b). According to Greglist database,^53^ there are two putative G4 DNA motifs locating at the sense strand 120 bp (Kit-G4-120) and 863 bp (Kit-G4-863) upstream of the transcriptional start site of mouse *c-kit* gene (Figure 7a). We first investigated whether these motifs could form G4 structures by using circular dichroism (CD) analysis and validated that these two G4 DNA motifs did form G4 structure. Mutation of the G4 DNA motifs disrupted G4 structure *in vitro* (Figure 7b). Taken together, there are two authentic G4 DNA structures on the promoters of mouse *c-kit* genes. Actually previous studies have demonstrated that DNA quadruplex structure exists on the *c-kit* promoter.^54, 55^ The previously validated G4 DNA structure is the Kit-G4-120 in this study, which conservatively exists on the promoters of mouse and human *c-kit*. Here we found another G4 DNA motif on mouse *c-kit* promoter. We used the G4 structure antibody BG4 to investigate whether G4 DNA structures (Kit-G4-120 and Kit-G4-863) existed on the *c-kit* promoters *in vivo* by chromatin immunoprecipitation (ChIP). Our results further confirmed that *c-kit* had two G4 DNA structures on the promoters of *c-kit in vivo*. GAPDH was used as the negative control (Figure 7c).

Although G4 DNA structures exist on *c-kit* promoter, it is unknown which factor can bind the motif and resolve the G4 structure to regulate *c-kit* expression. Our previous results suggested that RHAU might be one of the candidates. Therefore, we performed gel-shift assay to demonstrate the direct interaction. First, we investigated whether the wild-type G4 DNA structures bound with the purified GST-RHAU fusion protein. As expected, the gel-shift results showed these two G4 DNA structures directly bound to GST-RHAU, confirming these G4 DNA structures were bound by RHAU (Figure 7d). In the presence of ATP, RHAU had the resolvase activity on the G4-structures (Figure 7e). Further, we explored whether this binding was related to the *c-kit* transcriptional regulation. We cloned the fragment containing *c-kit* promoter harboring wild-type and mutated G4 DNA motifs to the upstream of reporter gene, luciferase (Figure 7f). Then, luciferase assay was performed. Reporter plasmids, Renilla control plasmid, and RHAU expression plasmid were co-transfected into HeLa cells. Twenty-four hours after transfection, the activity of report gene was detected. The results showed that the co-transfected RHAU dramatically increased luciferase activity (Figure 7g). These results indicated that RHAU bound directly to G4 DNA motif on the promoter region of *c-kit* and unwound the G4 DNA structure to enhance *c-kit* expression.

As mentioned above, the G4 DNA motifs on the *c-kit* promoter are conserved in mouse and human. Therefore, we raise a question that whether the change of RHAU level would alter *c-kit* expression in human cell lines. We observed RHAU overexpression in HEK 293T cells increased *c-kit* mRNA and protein level (Figures 7h and i). In contrast, knockdown of RHAU with specific siRNAs decreased *c-kit* mRNA and protein levels (Figures 7j and k). Thus, RHAU also can regulate *c-kit* expression in human cells, suggesting a conserved regulation machinery.

In summary, germ cell-specific RHAU ablation disrupts spermatogonia differentiation, which may be caused by, or at least partially caused by, the failure of unwinding the G4 structures in c-kit promoter by RHAU.

## Discussion

RHAU ablation leads to the blockage of spermatogonia differentiation. RHAU is a highly conserved gene in the vertebrate, and critical for embryonic development and hematopoiesis.^31, 33^ In this paper, we showed RHAU ablation in germ cells by crossing floxed RHAU mice with Vasa-Cre mice resulted in azoospermia. The abnormality begins with the blockage of spermatogonia differentiation, and subsequent meiosis initiation arresting. According to previous studies, G4 DNA structures are dynamically regulated in different phases of a cell cycle and show a relatively high level in the S phase.^6^ Our results show a higher level of cell proliferation at P7 and P8 when compared with P6 (Figures 5a and b), whereas P7 and P8 are the most important period for the first wave of spermatogonia differentiation and meiosis initiation. Considering all these evidences, we conclude that RHAU ablation alters the G4 DNA structure content in cell cycle of differentiating spermatogonia and thus triggers cell apoptosis and low cell proliferative activity, eventually disrupting the progression of meiosis.

Previous studies demonstrate that RHAU unwinds the G4 structure of DNA and RNA, and promotes the degradation of AU-rich mRNA,^19, 56, 57, 58^ regulating protein expression at both transcriptional and post-transcriptional levels. Undoubtedly, the integration of all these functions is essential for spermatogonia differentiation, meiosis initiation, and even meiosis progression. However, combining with the consideration of our energy and interest, the upstream transcription regulated by unwinding G4 DNA structure should be more important. Herein, we investigated the differential gene expression, which was induced mainly from G4 DNA. Indeed, RHAU deficiency increased the level of G4 DNA structure content and thus changed G4 DNA-related gene expression. Therefore, the function of RHAU unwinding the G4 DNA structures on the regulatory region of gene is essential for spermatogonia differentiation and meiosis initiation.

Downregulation of *c-kit* is a main cause of RHAU-mediated abnormal spermatogonia differentiation and meiosis. Spermatogonia differentiation and meiosis initiation are regulated by a large number of genes other than *c-kit* including, *Sohlh1*, *Sohlh2*, *Stra8*, *Bmp4*, and *Dazl.*^41, 59, 60, 61, 62, 63^ Among them, *Stra8* was downregulated in P7 and P8 RHAU ablation testes (Figure 3g and the upper panel of e), but *Stra8* is not a G4 DNA-related gene; *Bmp4*, *Sohlh1*, and *Dazl* show no significant change (Supplementary Figure 2A); *Sohlh2* was upregulated (Supplementary Figures 2A and B). Thus, the downregulation of *c-kit* may be the main source of deficiencies seen in spermatogonia differentiation mediated by RHAU knockout. Most importantly, the regulation of *c-kit* by RHAU was also exhibited in human cells, indicating this regulatory event was conserved among different species. In addition, *c-kit* is essential for the differentiation of multiple stem cells and acts as a oncogene in some types of cancer cells, suggesting it is possible that RHAU is involved in not only the controlling of spermatogonia differentiation, but also other types of stem cell differentiation and tumorigenesis.

Undifferentiated spermatogonia were not affected in germ cell-specific RHAU deletion. G4 DNA is usually located at the promoter of oncogenes.^64^ These genes are apt to promote cell proliferation and anti-apoptosis. Their deficiency often leads to developmental defects or insufficient cell proliferation. Interestingly, herein RHAU deletion affected the proliferation of differentiating but not undifferentiated spermatogonia. A possible explanation is that although the proliferation of undifferentiated spermatogonia may be affected, those beings initially assigned to differentiating spermatogonia was reprogrammed to supplement undifferentiated spermatogonia population because of decreased *c-kit* expression.

In conclusion, the dynamic formation and unwinding of G4 DNA structures are vital for spermatogenesis. RHAU regulated a large number of genes by unwinding the G4 DNA structures to control spermatogonia differentiation. *c-kit*, the direct target of RHAU, was dramatically downregulated in RHAU deletion mouse testes, which contributed to a spermatogonia differentiation blockage. However, the blockage of spermatogonia differentiation was incomplete and thus a portion of spermatogonia entered into meiosis but eventually stopped at the pachytene stage, indicating RHAU has a further role in pachytene spermatocytes differentiation. The role of RHAU in normal spermatogenesis during the pachytene stage requires a further exploration.

## Materials and Methods

### Generation of germ cell-specific RHAU knockout mice

Testicular germ cell-specific RHAU knockout mice were obtained by crossing RHAU flox/flox mice^33^ to Vasa-Cre line. Animal studies were carried out in an SPF animal facility accredited by the Association for the Assessment and Accreditation of Laboratory Animal Care, and all animal protocols were approved by the Animal Care and Use Committee of the Model Animal Research Center, the host for the National Resource Center for Mutant Mice in Nanjing University, China. Genotype analyses were performed by PCR. The PCR primers were listed in Supplementary Table 1.

### Quantitative RT-PCR

Testes were stripped of the tunica albuginea, placed in TRIzol (Invitrogen, Carlsbad, CA, USA), and stored at −20 °C. Total RNAs were prepared conforming to the manufacturer's protocol, and then DNase-treated using DNA Free Turbo (Ambion, Grand Island, NY, USA). One microgram of total RNA was reversely transcribed by using a RETROscript Kit (Ambion). qPCR was performed through the use of SYBR Green Core PCR Reagents (Applied Biosystems, Shanghai, China) on an ABI9700 Fast Real-time PCR machine (Applied Biosystems). Results were analyzed through the use of the Δ-Δ Ct method, and using GAPDH as the normalization control. qPCR primer sequences were listed in Supplementary Table 1.

### Antibodies

The following antibodies were used at the indicated concentration and obtained from the indicated sources: rabbit anti-MVH (1 : 500, Abcam, ab13840); rabbit anti-RHAU (1 : 100, Protein Technologies, Inc., Tucson, AZ, USA, 13159-1-AP); rabbit anti-RHAU (1 : 200, Abcam, Shanghai, China, ab70269); rabbit anti-phosphor-histone-H2A.X (1 : 200, CST, Danvers, MA, USA, #9718); rabbit anti-Sycp3 (1 : 200, Abcam, ab15093); mouse anti-MLH1 (1 : 50, BD Pharmingen, Shanghai, China, 551092); mouse anti-PLZF (1 : 200, Santa Cruz Biotechnology, Inc., Dallas, TX, USA, sc-28319); rabbit anti-Lin28 (1 : 500, Santa Cruz Biotechnology, Inc., sc-67266); rabbit anti-c-kit (1 : 300, CST, #3074); mouse anti-*β*-actin (1 : 10000, Santa Cruz, Biotechnology, Inc., sc-81178); mouse anti-BrdU (1 : 100, EMD Millipore, Billerica, MA, USA, 05–633); goat anti-rabbit IgG (H+L)-HRP (1 : 5000, Bioworld, St. Louis Park, MN, USA, BS10350); goat anti-mouse IgG-HRP (1 : 5000, Abmart, Shanghai, China, #M21002); Cy3-anti-mouse IgG (1 : 200, Jackson Immunoresearch, West Grove, PA, USA, 115-165-146); FITC-anti-rabbit IgG (1 : 200, Jackson Immunoresearch, 111-095-144); Cy3-anti-rabbit IgG (1 : 200, Jackson Immunoresearch, 111-165-003); and FITC-anti-mouse IgG (1 : 200, Jackson Immunoresearch, 115-095-146).

### Histological analysis and immunofluorescence

The testes were fixed with 4% paraformaldehyde at 4 °C overnight and processed for the paraffin sections, which were made from each recipient testis with an interval of 4 *μ*m between sections. All sections were stained with H&E. The paraffin sections were washed by a series of ethanol in different concentrations; boiled with 10 mM sodium citrate buffer (pH 6.0) for 15 min using a microwave; washed three times in phosphate-buffered saline (1 × PBS); transferred to the blocking solution containing 5% donkey serum or goat serum in 0.1% Tween/PBS for 1 h; and incubated with primary antibodies at 4 °C overnight. After all these steps, the secondary antibodies with 4',6-diamidino-2-phenylindole (DAPI) were added to the paraffin sections; and these sections were incubated for 45 min at room temperature. Then, slides were mounted, and images were captured using a FV1000 Olympus fluorescence microscope (Olympus Corporation, Tokyo, Japan).

### 5-Bromo-2-deoxyuridine (BrdU) incorporation

Mice were injected with 0.1 mg/g BrdU (BrdU/body weight), which was at a stock concentration of 10 g/l (Sigma, St. Louis, MO, USA, cat no B5002) in PBS. The testes were dissected, fixed, processed in ethanol, embedded with paraffin, and sectioned 2 h later than the intra-peritoneal injection. The following steps were the same as described for immunofluorescence.

### Apoptosis assay

Testes were fixed in 4% paraformaldehyde at 4 °C overnight, washed in PBS, dehydrated, paraffin embedded, and sectioned. Then, the detection of apoptosis at single-cell level was performed by the TdT *in situ* apoptosiss detection kit-DAB TUNEL-based apoptosis detection assay (R&D, cat no 4810-30-K) following manufacturer's instructions.

### Transformation and purification of BG4 protein

The plasmid expressing BG4 protein obtained from Professor Shankar Balasubramanian of University of Cambridge^6^ was transformed into *Escherichia coli* and then amplified during the cultivation. The cell culture was centrifuged for 30 min at 4 °C at 4000 × *g*, and re-suspended with the pellet in TES (50 mM Tris/HCl, pH 8.0; 1 mM EDTA, pH 8.0; 20% sucrose, filter, stored at 4 °C). The sample was centrifuged for 10 min for 8000 × *g* at 4 °C, rotated for 1 h at room temperature (RT) with Nickel affinity/anti-his tag beads, and purified on column.

### Immunohistochemistry

The paraffin sections were prepared as described above. The sections were de-waxed in xylenes, re-hydrated, and boiled for 10 min in 10 mM sodium citrate buffer (pH 6.0) using a microwave. Most primary antibodies, such as anti-RHAU, were treated with the sodium citrate, whereas anti-BG4 with trypsion pretreatment (25 mg Trypsin, 314 mg CaCl_2_.2 H_2_O in 100 ml 50 mM Tris, PH7.8) incubated for 20 min at 37 °C to prevent G4 DNA forming at an artificial state. After treating with 0.3% hydrogen peroxide for 10 min, these paraffin sections were incubated with blocking solution (TBST/5% normal goat serum). Then, we washed away the blocking solution, added primary antibodies, and washed the samples three times in Tris-borate-EDTA buffer (shorted for TBS). Next, biotinylated second antibodies were added to the samples, and ABC reagent (Vector ABC kit, PK-6100) was prepared conforming to the manufacturer's instructions. Then, DAB reagent (Vector DAB kit, SK-4100) was used to stain the slides. Eventually, the slides were stained with hematoxylin following the manufacturer's instructions.

### Western blotting analysis

Testes at different stages were collected and ground on ice in the lysis buffer (1% NP-40, 1% sodium deoxycholate, 0.1% SDS, 150 mM sodium chloride, 2 mM EDTA, 10 mM sodium phosphate, pH 7.2), which was supplemented with protease inhibitor cocktail (Roche, Shanghai, China) and 1 mM phenylmethylsulfonyl fluoride. Protein concentration was determined by the Bio-Rad DC protein assay (Bio-Rad, Life Science Research, Hercules, CA, USA). Equal amounts of total protein were separated on denaturing polyacrylamide gel and transferred to polyvinylidene difluoride transfer membrane (Bio-Rad). Blots were probed with the primary and appropriate secondary antibodies and detected using ECL chemiluminescence (GE Healthcare, Pittsburgh, PA, USA).

### Gene expression analysis

Total RNAs were prepared from mice testes as described.^65^ For microarray analysis, RNAs were labeled and hybridized to GeneChip Mouse Genome 430 2.0 arrays following the Affymetrix protocols. Data were analyzed by GeneSpring GX10. Significant genes were selected based on the criteria that *P*-values were smaller than 0.05 and the fold changes were greater than 1.5. For enrichment analysis of biological process ontology, probe lists were analyzed in DAVID^64^ and processes were selected if *P*-values were smaller than 0.05.

### Quadruplex formation and CD spectropolarimetry

A CD study was performed as previously described.^66^ Briefly, quadruplex formation was carried out by dissolving synthetic DNA in ddH_2_O at a concentration of 100 mM. A total 20 *μ*l DNA was mixed with 180 *μ*l of TE buffer (10 mM Tris-HCl, 0.1 mM EDTA, pH 7.5) and incubated in a PCR thermocycler at 98 °C for 10 min and then held at 80 °C. KCl was added immediately to a final concentration of 50 mM; and the solution was allowed to cool down slowly to the room temperature. CD spectra were recorded on a spectropolarimeter (JASCO J-715), using a quartz cell of 1.0 mm optical path length, over a wavelength range from 200 to 350 nm at 25 °C with 1 nm increments and an averaging time of 2 s

### ChIP assays

ChIP assays were performed as previously described,^67, 68^ with some noteworthy changes. Testes were dissected from adult male mice (P35) and tunica albuginea was/were removed. Tissues were minced with scissors into 1 mm^3^ pieces in PBS. After washing twice with PBS to remove lumen fluid and sperm, the tissue pieces were crosslinked in 1% formaldehyde at the room temperature for 10 min. The tissue pieces were then pelleted, rinsed, and resuspended in PBS, which contained protease inhibitors, as well as ground on ice using a glass homogenizer. Nuclei were filtered, collected, resuspended in lysis buffer (1% SDS, 10 mM EDTA, 50 mM Tirs-HCl, pH 8.1, 1 H protease inhibitor cocktail), and sonicated to yield 200–300 bp DNA fragments. After centrifuging for 10 min, supernatants were collected. Then, 30 μl of the supernatants were used as input. The remainder was diluted twofold in the dilution buffer (1% Triton X-100, 2 mM EDTA, 150 mM NaCl, 20 mM Tris-HCl, pH 8.1) and subjected to immunoprecipitation overnight at 4 °C with 4 μg specific antibody. After a 5- to 6-h incubation with Flag-beads (Sigma, A2220) and 2 h incubation with Protein A beads (Santa Cruz, CA, sc-2001), chromatin–antibody–bead complexes were washed sequentially twice with TSE I (0.1% SDS, 1% Triton X-100, 2 mM EDTA, 20 mM Tris-HCl, pH 8.1, and 150 mM NaCl), twice with TSE II (0.1% SDS, 1% Triton X-100, 2 mM EDTA, 20 mM Tris- HCl, pH 8.1, and 500 mM NaCl), twice with buffer III (0.25 M LiCl, 1% Nonidet P-40, 1% deoxycholate, 1 mM EDTA, 10 mM Tris-HCl, pH 8.1), and twice with TE buffer (1 mM EDTA and 10 mM Tris-HCl, pH 8.1). Chromatin was eluted with elution buffer (1% SDS and 0.1 M NaHCO_3_) before the reversal of crosslinks with Proteinase K (Invitrogen) at 65 °C for at least 8 h. DNA was purified using phenol-chloroform extraction and ethanol precipitation, and resolved in optimal volume of double-distilled H_2_O. The specific antibody BG4 with flag-tag was purified in our laboratory, and the protocol of transformation and purification of this protein had been described in detail. DYKDDDDK Tag antibody was purchased from CST (Cat. #2368 S). The primers for ChIP are listed in the Supplementary Table1.

### Luciferase assay

HeLa cells cultured in 96-well plates were transfected with 100 ng of the reporter constructs, which contained the *c-kit* promoter with the potential G4 structure-forming sequences and 50 ng of a control plasmid pRL-TK (expressing Relina luciferase). To detect the effect of RHAU on the *c-kit* promoter, 100 ng of RHAU expression plasmid or empty vector were co-transfected. Aliquots of medium from the transfected wells were collected 24 h post transfection to measure luciferase activity, and normalized against the Relina activity in the same sample following the procedure previously described.^69^ Each condition was tested in triplicate and repeated over three times.

### 5′-^32^P-end labeled of G4 nucleic acids and G4 nucleic acid structures formation

The annealed G4 oligonucleotide was incubated with T4 polynucleotide kinase and *γ*-^32^P-ATP for 30 min at 37 °C to produce a 5′-^32^P-end labeling of G4 oligonucleotide. Then, the labeled nucleic acids were purified by a MicroSpin G25 column (GE healthcare) and equilibrated with the TEK buffer. More detailed information was the same as previously described.^66^

The method of the formation of G4 nucleic acids was referred as the previous reports.^70^ The labeled G4 nucleic acids solution was added into tubes and incubated in a thermocycler at 98 °C for 10 min, and then at 80 °C. Meanwhile, EDTA of pH 8.0 was aliquoted into these tubes to ensure that the final concentration was 25 mM. Then the temperature was gradually and slowly decreased to the room temperature. Eventually, 5′-^32^P-end labeled of G4 nucleic acids were stored at 4 °C for 2–3 days.

### Purification of RHAU protein and gel shift

The procedure of transformation and purification of RHAU was the same as the previously described BG4. Gel shift assay was performed as previously described.^66^ Recombinant RHAU purified at concentration of 1 *μ*M was incubated with 1 pM of 5′-^32^P-labeled G4 nucleic acid in the RES-EDTA buffer (100 mM KCl, 10 mM NaCl, 3 mM MgCl_2_, 50 mM Tris-acetate, pH 7.8, 70 mM glycine, 10% glycerol, 10 mM EDTA) at 37 °C for 30 min. Bound mixtures were then analyzed by 10% non-denaturing polyacrylamide gel. Electrophoresis was performed at 100 V for 2 h in a cold room. Gels were imaged on a Typhoon 9210 Imager (GE Healthcare).

To demonstrate the effect of ATP on RHAU/Kit association, we referred the previously described method.^56^ 1 *μ*M RHAU was incubated with 1 pM 5′-^32^P-labeled self-annealed kit in the presence or absence of 5 mM ATP at 37 °C for 30 min, respectively. All the samples were analyzed on 10% non-denaturing polyacrylamide gel at 150 V for 2 h. The following steps were the same as the procedure described above.

### Statistical analyses

Statistical analyses were performed with Excel 2002 software (Microsoft, Redmond, WA, USA). A mean value was shown with a standard error. Student's *t*-test was used for the comparison of two independent groups (*P<0.05, **P<0.01, *** *P*<0.001).

## Abbreviations

G4 DNA: G-quadruplex DNA
RHAU: RNA helicase associated with AU-rich element
H&E: hematoxylin and eosin
BrdU: 5-Bromo-2-deoxyuridine
CD: circular dichroism
ChIP: Chromatin immunoprecipitation
RT-qPCR: reverse transcription and quantitative PCR
TUNEL: terminal deoxynucleotidyl transfer dUTP nick end labeling
SSC: spermatogonia stem cell
PBS: phosphate-buffered saline
