# Supplementary material for: A G-quadruplex DNA structure resolvase, RHAU, is essential for spermatogonia differentiation
Source: Cell Death Dis. 2015 Jan 22;6(1):e1610–. doi: 10.1038/cddis.2014.571 (PMC4669769; doi:10.1038/cddis.2014.571)
Supplement: Supplementary Table 1 [file cddis2014571x1.doc]

**Supplementary Table 1 Primers for genotyping, Q-PCR and CHIP**

| **Primers for genotyping** | **Sequence (5' to 3')** |
| --- | --- |
| RHAU forward | CTGCGTAGGGTAGCTTATG |
| RHAU reverse | ATCCGACTGTAGATTCCTTT |
| Vasa forward | GCCTGCATTACCGGTCGATGC |
| Vasa reverse | CAGGGTGTTATAAGCAATCCC |
|  |  |
| **Primers for Q-PCR** | **Sequence (5' to 3')** |
| RHAU forward: | ATGGATGAACGTCGAGAAGAGC |
| RHAU reverse: | ATACCCATGATCCTCAGGAGC |
| Spo11 forward: | CGTGGCCTCTAGTTCTGAGGT |
| Spo11 reverse: | GCTCGATCTGTTGTCTATTGTGA |
| Dmc1 forward: | CCCTCTGTGTGACAGCTCAAC |
| Dmc1 reverse: | GGTCAGCAATGTCCCGAAG |
| Hormad1 forward: | GGCTCCTAGCTGTTTCAGTATCT |
| Hormad1 reverse: | TTGTCCCATAAGCACGTTCTG |
| Stra8 forward: | ACAACCTAAGGAAGGCAGTTTAC |
| Stra8 reverse: | GACCTCCTCTAAGCTGTTGGG |
| *c-kit* forward: | GGCCTCACGAGTTCTATTTACG |
| *c-kit* reverse: | GGGGAGAGATTTCCCATCACAC |
| PLZF forward: | GAGACACACAGACAGACCCATACT |
| PLZF reverse: | CACACATAACACAGGTAGAGGTACG |
| GFR α forward: | TGCGTATCTACTGGAGCATGT |
| GFR α reverse: | GGGGAGAGATTTCCCATCACAC |
| GAPDH forward: | CATGGCCTTCCGTGTTCCT |
| GAPDH reverse: | GCGGCACGTCAGATCCA |
|  |  |
| **Primers for CHIP** | **Sequence (5' to 3')** |
| *c-kit* site120 forward: | TTGCTCCTAAGGCAGACCACA |
| *c-kit* site120 reverse: | GCATTTGGGTGCCTTTTCTC |
| *c-kit* site863 forward: | GGGATCAGCTTATTGCAGCC |
| *c-kit* site863 reverse: | CTCCAGCCCTCCTCCCAG |
| GAPDH forward: | TGGCAAAGTGGAGATTGTTGCC |
| GAPDH reverse: | AAGATGGTGATGGGCTTCCCG |
