# Supplementary figures and images for: A G-quadruplex DNA structure resolvase, RHAU, is essential for spermatogonia differentiation
Source: Cell Death Dis. 2015 Jan 22;6(1):e1610–. doi: 10.1038/cddis.2014.571 (PMC4669769; doi:10.1038/cddis.2014.571)

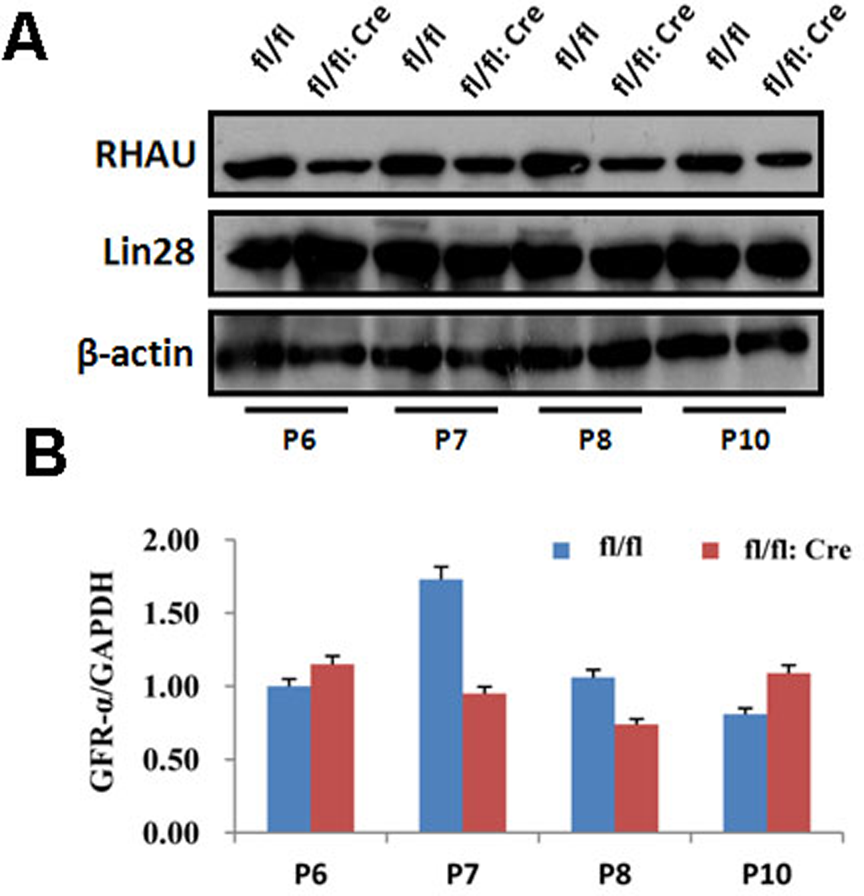

Supplement: Supplementary Figure 1 [file cddis2014571x2.tif]

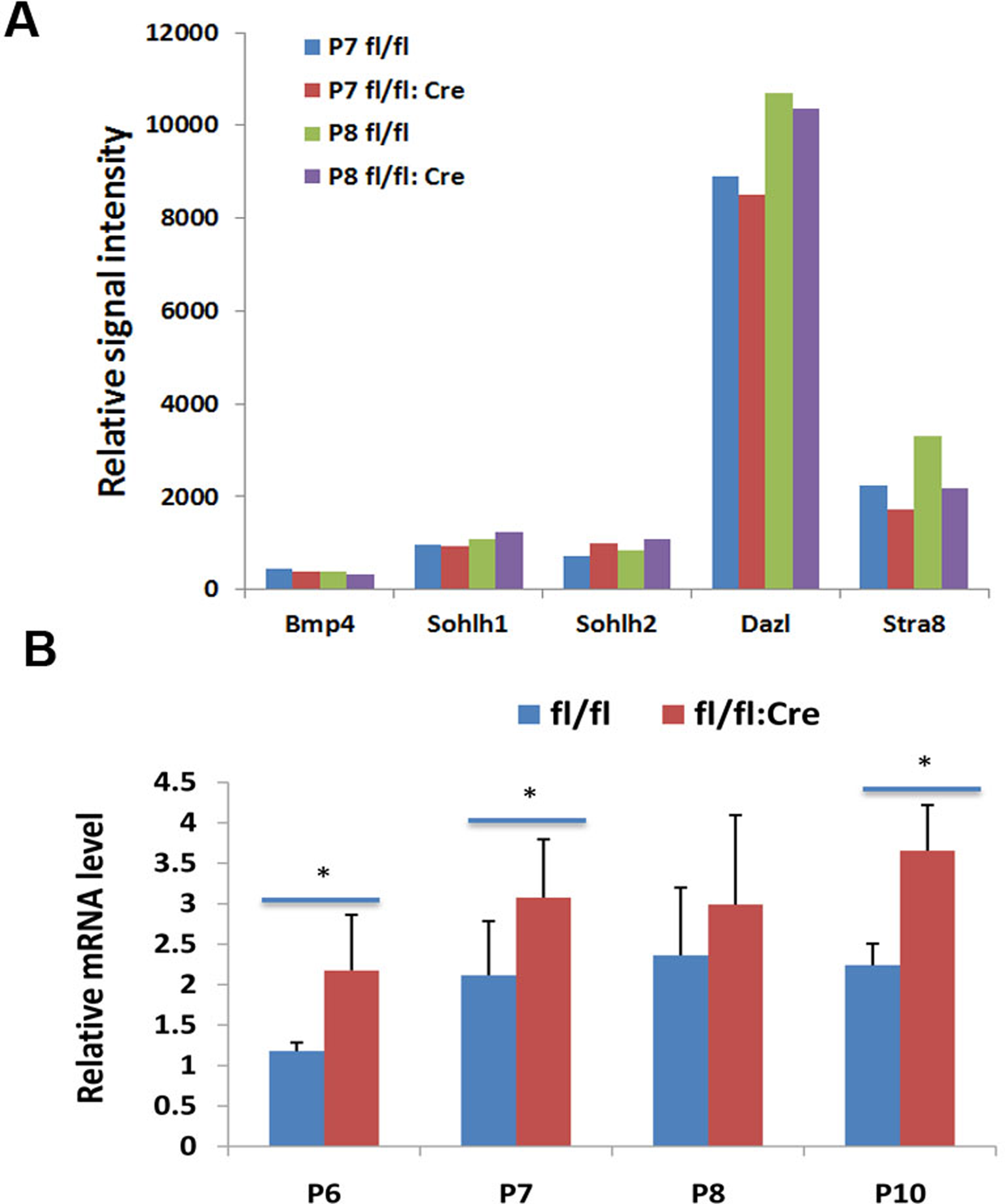

Supplement: Supplementary Figure 2 [file cddis2014571x3.tif]
