# Supplementary material for: A G-quadruplex DNA structure resolvase, RHAU, is essential for spermatogonia differentiation
Source: Cell Death Dis. 2015 Jan 22;6(1):e1610–. doi: 10.1038/cddis.2014.571 (PMC4669769; doi:10.1038/cddis.2014.571)
Supplement: Supplementary Figure Legends [file cddis2014571x4.doc]

**Supporting information**

**Fig. S1 Some marker genes related with SSC development were not affected in germ-cell specific RHAU knockout mice**

1. Western blotting analysis of Lin28 expression in the whole testes of P6, P7, P8, and P10 control and RHAU deletion mice.
2. Quantitative-PCR analysis of GFR-α expression in the whole testes of P6, P7, P8, and P10 control and RHAU deletion mice (mean±S.D., n=3).

**Fig. S2 Analysis of meiosis related genes**

1. The microarray analysis of the relative expression of *Bmp4*, *Sohlh1*, *Sohlh2*, *Dazl*, and *Stra8* in testes from P7 and P8 control and RHAU deletion mice.
2. Quantitative-PCR analysis of *Sohlh2* expression in testes from P6, P7, P8, and P10 control and RHAU deletion mice (mean±S.D., n=3). The relative expression level of *Sohlh2* mRNA normalized to GAPDH (* p<0.05).
